# Postoperative survival of pulmonary invasive mucinous adenocarcinoma versus non-mucinous invasive adenocarcinoma

**DOI:** 10.1186/s12890-023-02305-x

**Published:** 2023-01-09

**Authors:** Dongyu Cui, Shaonan Xie, Qingyi Liu

**Affiliations:** Department of Thoracic Surgery, The Fourth Hospital of Hebei Medical University, Shijiazhuang, China

**Keywords:** Mucinous adenocarcinoma, Prognosis, Propensity score matching, NSCLC, Risk factor

## Abstract

**Purpose:**

In 2015, the World Health Organization renamed mucinous bronchioloalveolar adenocarcinoma as pulmonary invasive mucinous adenocarcinoma (IMA). Due to its low incidence and unclear prognosis with surgical treatment, previous studies have presented opposing survival outcomes. We aimed to investigate the differences in surgical prognosis and prognosis-related risk factors by comparing IMA with non-mucinous invasive adenocarcinoma (NMA).

**Methods:**

A total of 20,914 patients diagnosed with IMA or NMA from 2000 to 2014 were screened from the Surveillance, Epidemiology, and End Results database. The screened patients were subjected to propensity score matching (PSM) in a 1:4 ratio to explore the survival differences between patients with IMA and NMA and the factors influencing prognosis.

**Results:**

For all patients, IMA was prevalent in the lower lobes of the lungs (*p* < 0.0001), well-differentiated histologically (*p* < 0.0001), less likely to have lymph node metastases (94.4% vs. 72.0%, *p* < 0.0001) and at an earlier pathological stage (*p* = 0.0001). After PSM, the IMA cohort consisted of 303 patients, and the NMA cohort consisted of 1212 patients. Kaplan‒Meier survival analysis showed no difference in overall survival (OS) between patients in the IMA cohort and those in the NMA cohort (*p* = 0.7). Cox proportional hazards analysis showed that differences in tumor pathological type did not influence OS between the two cohorts (*p* = 0.65). Age (HR: 1.98, 95% CI 1.7–2.31, *p* < 0.0001), gender (HR: 0.64, 95% CI 0.55–0.75, *p* < 0.0001), and radiation treatment (HR: 2.49, 95% CI 1.84–3.37, *p* < 0.0001) were independent predictors of patient OS.

**Conclusion:**

There was no significant difference in OS between patients with IMA and those with NMA after surgical treatment. Age, sex, and radiation treatment can independently predict OS.

## Introduction

According to Global Cancer Statistics 2021 [1], there were approximately 19.3 million new tumor cases and approximately 10 million deaths worldwide, with approximately 2.2 million new cases and 1.7 million deaths from lung cancer, which remains the deadliest cancer to date. The most common type of lung cancer today is lung adenocarcinoma, which accounts for approximately 50% of all lung cancers [2]. A specific subtype of lung adenocarcinoma, once known as mucinous bronchioloalveolar adenocarcinoma [3], accounts for approximately 5% of all adenocarcinomas of the lung [4]. Because there are many subtypes of bronchoalveolar carcinoma, the prognosis of each subtype varies greatly. In 2011, the International Association for the Study of Lung Cancer (IASLC), the American Thoracic Society (ATS), and the European Respiratory Society (ERS) jointly proposed renaming mucinous bronchioloalveolar adenocarcinoma as invasive mucinous adenocarcinoma (IMA) [5]. In 2015, the WHO officially updated the classification of lung tumors and renamed mucinous bronchioloalveolar adenocarcinoma as IMA.

IMA possesses unique pathological and molecular biological features. The tumor cells are composed of goblet cells and columnar cells rich in mucin. Compared to other types of lung adenocarcinoma, the cellular heterogeneity of IMA is less pronounced, making it more challenging to diagnose clinically [4, 6]. On imaging, IMA may appear as either a nodule or a mass or as a pneumonia-like feature [7]. The diagnosis of IMA can therefore be easily missed in clinical practice based on imaging data alone. At the genetic level, previous studies have demonstrated that IMA is susceptible to mutations in the KRAS gene [8], accounting for approximately 35–75% of all mutation types [9, 10]. The prognosis of patients with IMA with mutations is poor; the prognosis of IMA is highly variable, and previous studies have shown contrasting survival outcomes. Some studies have shown a significantly better overall survival (OS) than non-mucinous invasive adenocarcinoma (NMA) [11–13]. Other reports have shown conflicting results [14–16]. The relationship between the clinicopathological features and prognosis of IMA is poorly understood, as this type of adenocarcinoma is rare, and there are few retrospective studies of large numbers of cases. The accurate diagnosis of IMA is crucial to patient treatment and prognosis. Therefore, this study collected data from patients with pathologically confirmed IMA and NMA from 2000 to 2014 through the Surveillance, Epidemiology and End Results (SEER) database to compare whether there is a difference in OS between patients with IMA and NMA. This provides support for the clinical treatment of patients with IMA.

## Methods

### Patient selection

This study was a retrospective study in which we used SEER*Stat 8.4.0 software and selected data collected from 17 centers from 2000 to 2019 (SEER Research Plus Data, 17 Registries, Nov 2021 Sub[2000–2019]), resulting in 20,914 patients meeting our inclusion criteria. The inclusion criteria were as follows: (1) patients with a year of diagnosis of 2000–2014 were selected; (2) complete follow-up was available; (3) virtual survival status and exact survival time were available; (4) the site of diagnosis was “Lung and Bronchus”(5) “only one primary cancer” status was met; and (6) the pathological type at diagnosis was selected by “Site and Morphology, ICD-O-3 Hist/Behav, malignant”. The pathological types of NMA were as follows: adenocarcinoma (8140/3), bronchioloalveolar carcinoma nonmucinous (8252/3), papillary adenocarcinoma (8260/3), bronchioloalveolar NOS (8250/3), adenocarcinoma with mixed subtypes (8255/3), and acinar cell carcinoma (8550/3). The pathological type of IMA was bronchioloalveolar carcinoma mucinous (8253/3). In contrast, 8481/3 mucinous-producing adenocarcinoma is a mucinous-producing adenocarcinoma; this type is not IMA, so we excluded this pathological type.

The data were then screened, first selecting patients with a single primary tumor surgically removed and confirmed by pathology. Patients diagnosed by autopsy and puncture biopsy were excluded, and patients with an overall survival of less than one month were excluded. Patients with unknown age, race, marital status, radiotherapy information, or unknown survival status were also excluded. Patients with unknown lymph node dissection and distant metastatic status were excluded. Finally, we selected data from patients who did not develop metastases.

**Fig. 1:**
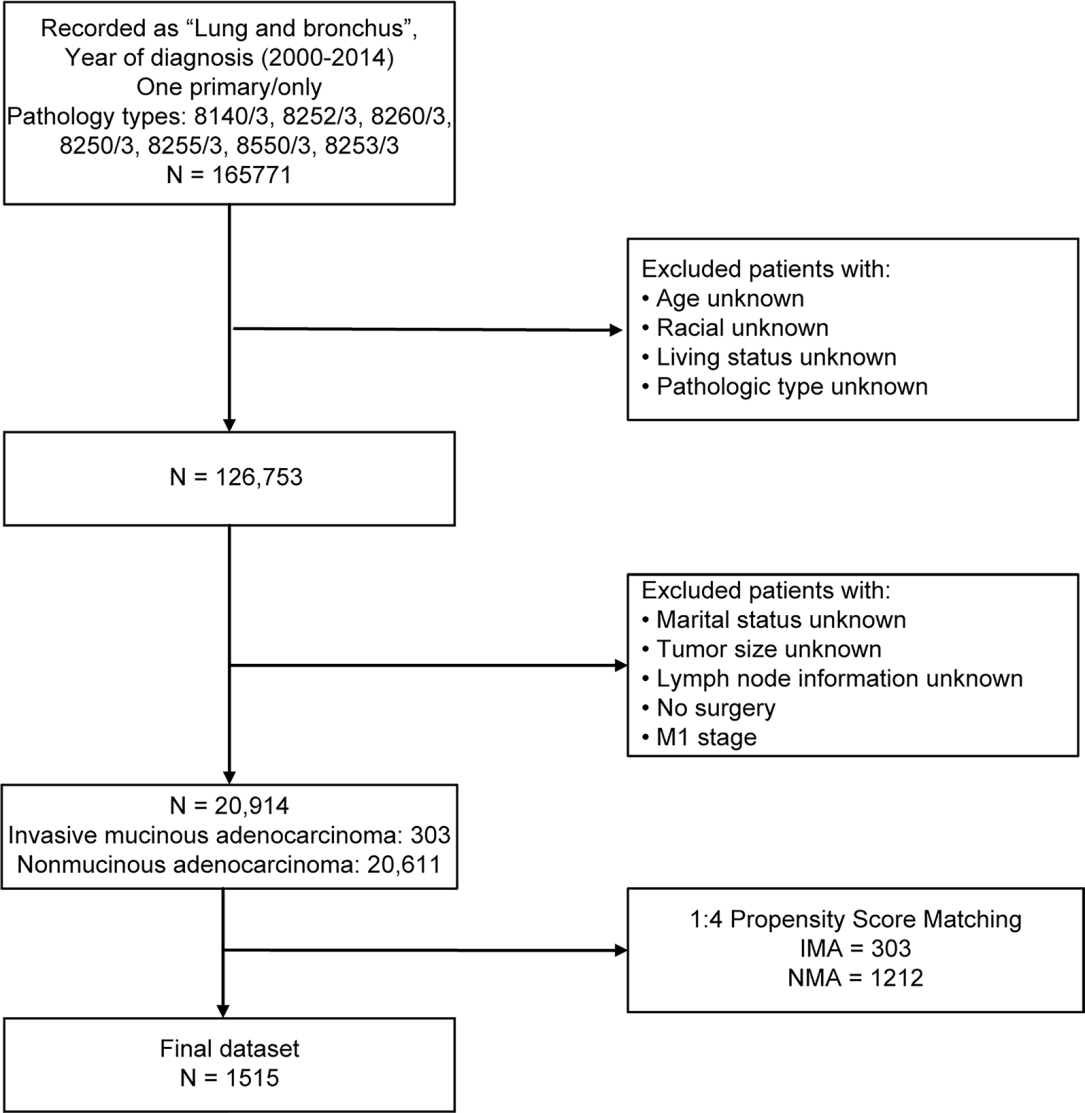
Screening process including reasons for exclusion from the dataset

Once the data had been selected, we extracted the patient details from the database, including basic demographic, TNM stage, differentiation grade, lesion location and size, survival time, survival status, radiotherapy, and chemotherapy information. Figure 1 summarizes the process of cleaning the data. A final dataset was created to describe the clinical characteristics of patients with IMA and NMA. This included 20,611 patients with NMA and 303 patients with IMA. To exclude the effect of confounding factors on survival outcomes, the screened patients were subjected to propensity score matching (PSM) with a ratio of 1:4.

### Statistical analysis

The primary endpoint of this study was the OS of patients in both cohorts, counted in months. Patients who survived at the last follow-up date in the SEER database (including those who died after the follow-up date) were recorded as surviving.

Characteristics were summarized using standardized statistical parameters, with continuous variables described as the mean ± standard deviation or median + upper and lower quartiles and categorical variables described as frequencies and percentages based on the results of the Kolmogorov‒Smirnov test. Analysis of the distribution of nonnormal continuous variables was performed using the Mann‒Whitney U test. Categorical variables were compared using the chi-square test or Fisher's exact test.

As the patients selected from the database all had TNM staging according to the 6th edition of the AJCC TNM staging system, we converted all patients' staging to the 8th edition TNM staging by comparing the 6th and 8th editions of the AJCC. The dataset was matched with 1:4 PSM using the MatchIt package of R software. The matched IMA cohort contained 303 patients, and the NMA cohort contained 1212 patients. The balance between the two groups was checked by standardized mean differences after matching.

The Kaplan‒Meier method was applied to obtain survival and OS data for patients with IMA and NMA. PH assumptions were made for all variables included in the model and all covariates pass the PH assumption. Cox PH analysis was used to evaluate the influencing factors affecting survival, and variables with *p* values < 0.05 in the univariate analysis were included in the multivariable analysis. They were evaluated again using the Cox PH analysis model with the propensity-matched data. All *p* values less than 0.05 were considered statistically significant. Hazard ratios (HRs) and 95% confidence intervals (CIs) were used to indicate the relative risk of each factor.

## Results

### General characteristics of IMAs and NMAs

A total of 20,914 eligible patients were included in this study. The baseline characteristics of patients with IMA and NMA are shown in Dataset 1 (Table 1). There were no differences in age at diagnosis (*p* = 0.299), race (*p* = 0.320), gender (*p* = 0.156), marital status (*p* = 0.029), right and left lung distribution (*p* = 0.134) or type of surgery (*p* = 0.138). However, the distribution of upper, middle, and lower lobes (*p* < 0.0001), tumor histological grade (*p* < 0.0001), T stage (*p* < 0.0001), N stage (*p* < 0.0001), pathological stage (*p* = 0.0001), tumor size (*p* < 0.0001), radiotherapy (*p* < 0.0001), and chemotherapy (*p* < 0.0001) showed differences between the two groups. The majority of tumor differentiation in IMA was grade I, while NMA showed a different distribution of differentiation. The TNM staging of patients in the SEER database was staged according to the 6th edition of the AJCC staging system. After conversion to the 8th edition staging system, the two groups showed differences in T stage, N stage, and TNM stage (*p* < 0.0001). Ninety-four percent of patients in the IMA group were lymph node metastasis free. The two groups also showed significant differences in the receipt of adjuvant radiotherapy and chemotherapy (*p* < 0.0001). There was no significant difference between the IMA group and the NMA group in terms of the choice of surgical procedure.

**Table 1:** Basic characteristics of patients with IMA and NMA at diagnosis

Variables	IMA	NMA	*p*
	303	20,611	
*Age (years)*			
≤ 65	142 (46.86%)	9044 (43.88%)	0.299
> 65	161 (53.14%)	11,567 (56.12%)	
*Sex*			
Male	116 (38.28%)	8727 (42.34%)	0.156
Female	187 (61.72%)	11,884 (57.66%)	
*Marital status*			
Single	75 (24.75%)	5504 (26.70%)	0.029
Divorced	27 (8.91%)	2770 (13.44%)	
Married	201 (66.34%)	12,337 (59.86%)	
*Race*			
Black	32 (10.56%)	1686 (8.18%)	0.32
Other	30 (9.90%)	2030 (9.85%)	
White	241 (79.54%)	16,895 (81.97%)	
*Location*			
Upper	102 (33.66%)	12,781 (62.01%)	< 0.0001
Lower	180 (59.41%)	6312 (30.62%)	
Middle	15 (4.95%)	1033 (5.01%)	
Other	6 (1.98%)	485 (2.35%)	
*Grade*			
Grade I	179 (59.08%)	3692 (17.91%)	< 0.0001
Grade II	51 (16.83%)	9436 (45.78%)	
Grade III	9 (2.97%)	6353 (30.82%)	
Grade IV	0 (0.00%)	130 (0.63%)	
Unknown	64 (21.12%)	1000 (4.85%)	
*Laterality*			
Left	135 (44.55%)	8307 (40.30%)	0.134
Right	168 (55.45%)	12,304 (59.70%)	
*T*			
T1	135 (44.55%)	13,448 (65.25%)	< 0.0001
T2	74 (24.42%)	4900 (23.77%)	
T3	44 (14.52%)	1443 (7.00%)	
T4	50 (16.50%)	820 (3.98%)	
*N*			
N0	286 (94.39%)	14,863 (72.11%)	< 0.0001
N1	9 (2.97%)	2888 (14.01%)	
N2	8 (2.64%)	2795 (13.56%)	
N3	0 (0.00%)	65 (0.32%)	
*Stage*			
IA1	19 (6.27%)	939 (4.56%)	0.0001
IA2	55 (18.15%)	5399 (26.19%)	
IA3	55 (18.15%)	4261 (20.67%)	
IB	44 (14.52%)	2105 (10.21%)	
IIA	27 (8.91%)	964 (4.68%)	
IIB	45 (14.85%)	3127 (15.17%)	
IIIA	55 (18.15%)	3229 (15.67%)	
IIIB	3 (0.99%)	569 (2.76%)	
IIIC	0 (0.00%)	18 (0.09%)	
Size (median [IQR])	35.000 [20.000, 60.000]	25.000 [18.000, 36.000]	< 0.0001
*Operation*			
Lobectomy	263 (86.80%)	16,849 (81.75%)	0.138
Lobectomy extended	7 (2.31%)	855 (4.15%)	
Other sublobar	3 (0.99%)	107 (0.52%)	
Pneumonectomy	6 (1.98%)	691 (3.35%)	
Segmentectomy	5 (1.65%)	565 (2.74%)	
Wedge	19 (6.27%)	1544 (7.49%)	
*Chemotherapy*			
No	254 (83.83%)	14,909 (72.34%)	< 0.0001
Yes	49 (16.17%)	5702 (27.66%)	
*Radiation*			
No	294 (97.03%)	18,256 (88.57%)	< 0.0001
Yes	9 (2.97%)	2355 (11.43%)	

### Comparative postoperative survival of patients with IMA and NMA: analysis of the propensity-matched dataset

**Fig. 2:**
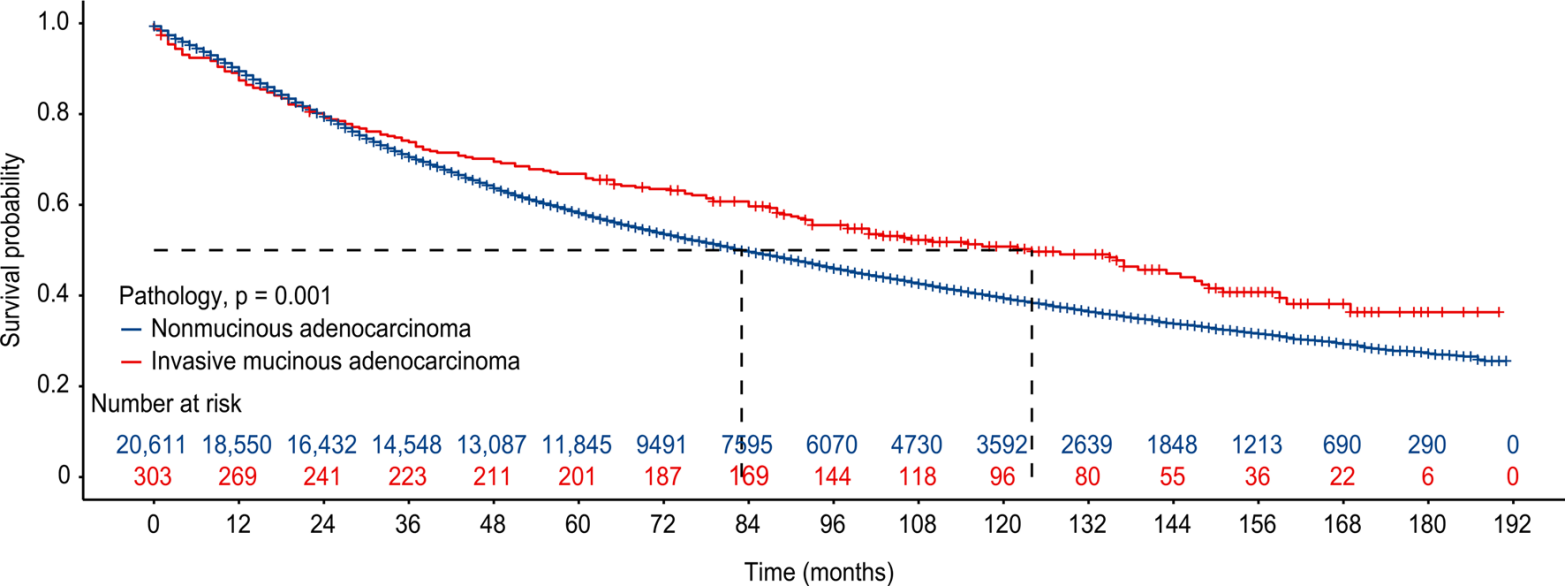
Kaplan‒Meier Survival curves for IMA patients and NMA patients before PSM

**Fig. 3:**
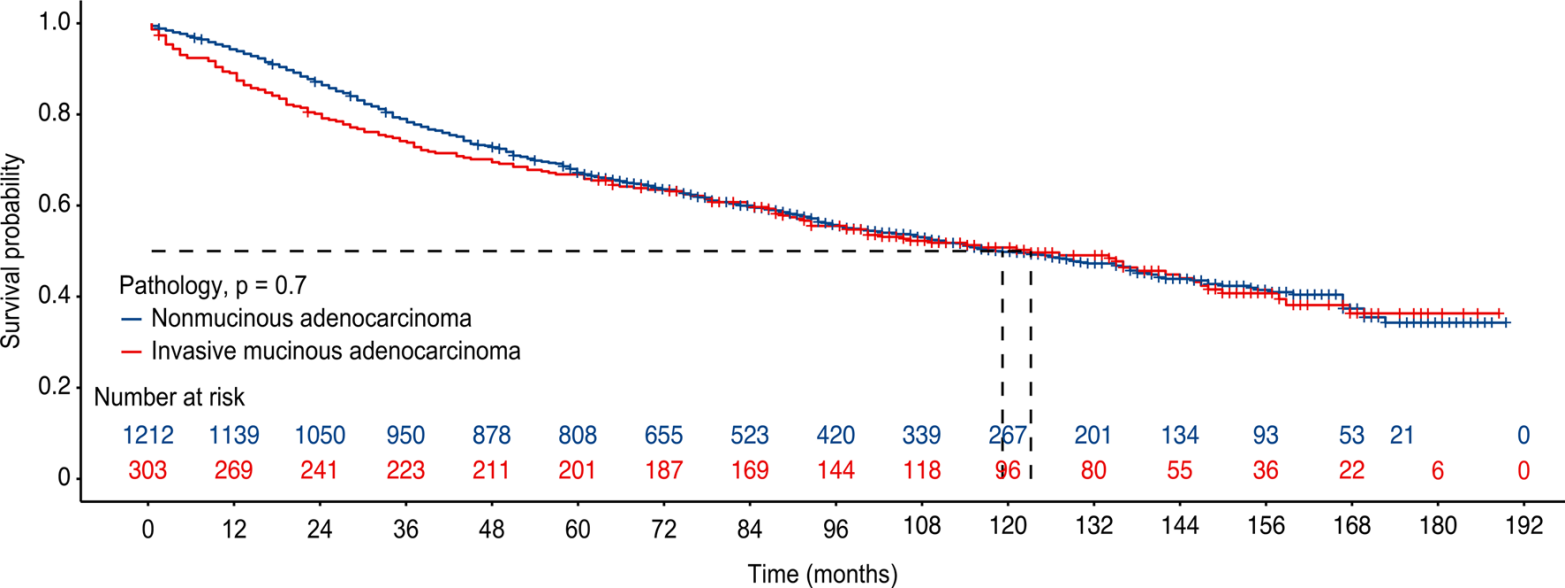
Kaplan‒Meier Survival curves for IMA patients and NMA patients after PSM

The screened patients were grouped in PSM according to a ratio of 1:4. This resulted in 303 patients with IMA and 1212 patients with NMA. The baseline characteristics of the two groups are shown in Dataset 2 (Table 2). There were no differences between the matched patients in variables such as age, race, gender, and tumor differentiation grade. This allowed for the exclusion of confounding factors on the survival of patients in both cohorts. In the prematching cohort, the median survival time was longer in the IMA group than in the NMA group (124 ± 34 months (95% Cl 96.23–151.77) for patients with IMA and 83 ± 30 months (95% Cl 80.82–85.18) for patients with NMA *p* = 0.001) (Fig. 2). However, patients with IMA and NMA in the matched cohort did not show a difference in survival. The median survival times were 124 ± 34 months (95% CI 96.23–151.77) for patients with IMA and 119 ± 44 months for patients with NMA (95% CI 105.47–132.54; *p* = 0.7) (Fig. 3). To further validate the impact of IMA and NMA on patient OS, univariate and multivariate Cox analyses were performed on the prematched and postmatched data (Tables 3 and 4). Variables that were significant in the univariate analysis were then subjected to multivariable analysis. In the unmatched data, Cox multivariable analysis showed that age, radiotherapy, chemotherapy, histological grade, location, marital status, mode of surgery, race, gender, tumor volume, T stage, N stage, and pathological stage could predict the OS of patients, while in the postmatched data, Cox multivariable analysis showed that age, gender, and receipt of radiotherapy independently predicted the OS of patients.

**Table 2:** Basic characteristics of patients with IMA and NMA after PSM

Variables	IMA	NMA	*p*
	303	1212	
*Age (years)*			
≤ 65	142 (46.86%)	570 (47.03%)	1
> 65	161 (53.14%)	642 (52.97%)	
*Sex*			
Male	116 (38.28%)	451 (37.21%)	0.730
Female	187 (61.72%)	761 (62.79%)	
*Marital status*			
Single	75 (24.75%)	275 (22.69%)	0.745
Divorced	27 (8.91%)	109 (8.99%)	
Married	201 (66.34%)	828 (68.32%)	
*Race*			
Black	32 (10.56%)	121 (9.98%)	0.464
Other	30 (9.90%)	95 (7.84%)	
White	241 (79.54%)	996 (82.18%)	
*Location*			
Upper	102 (33.66%)	426 (35.15%)	0.914
Lower	180 (59.41%)	698 (57.59%)	
Middle	15 (4.95%)	67 (5.53%)	
Other	6 (1.98%)	21 (1.73%)	
*Grade*			
Grade I	179 (59.08%)	731 (60.31%)	0.916
Grade II	51 (16.83%)	211 (17.41%)	
Grade III	9 (2.97%)	36 (2.97%)	
Unknown	64 (21.12%)	234 (19.31%)	
*Laterality*			
Left	135 (44.55%)	537 (44.31%)	0.938
Right	168 (55.45%)	675 (55.69%)	
*T*			
T1	135 (44.55%)	546 (45.05%)	0.157
T2	74 (24.42%)	348 (28.71%)	
T3	44 (14.52)	171 (14.11%)	
T4	50 (16.50%)	147 (12.13%)	
*N*			
N0	286 (94.39%)	1127 (92.99%)	0.684
N1	9 (2.97%)	45 (3.71)	
N2	8 (2.64%)	40 (3.30%)	
*Stage*			
IA1	19 (6.27%)	80 (6.60%)	0.671
IA2	55 (18.15%)	207 (17.08%)	
IA3	55 (18.15%)	232 (19.14%)	
IB	44 (14.52%)	214 (17.66%)	
IIA	27 (8.91%)	117 (9.65)	
IIB	45 (14.85%)	179 (14.77%)	
IIIA	55 (18.15%)	168 (13.86%)	
IIIB	3 (0.99%)	15 (1.24%)	
Size (median [IQR])	35.000 [20.000, 60.000]	34.000 [21.000, 52.250]	0.320
*Operation*			
Lobectomy	263 (86.80%)	1057 (87.21%)	0.977
Lobectomy extended	7 (2.31%)	34 (2.81%)	
Other sublobar	3 (0.99%)	10 (0.83%)	
Pneumonectomy	6 (1.98%)	20 (1.65%)	
Segmentectomy	5 (1.65%)	20 (1.65%)	
Wedge	19 (6.27%)	71 (5.86%)	
*Chemotherapy*			
No	254 (83.83%)	1012 (83.50%)	0.890
Yes	49 (16.17%)	200 (16.50%)	
*Radiation*			
No	294 (97.03%)	1162 (95.87%)	0.353
Yes	9 (2.97%)	50 (4.13%)

**Table 3:** Univariate and multivariate Cox proportional hazards analyses

Variables	Univariate analysis			Multivariable analysis		
	HR	95% CI	*p*	HR	95% CI	*p*
*Age (years)*						
≤ 66	Reference					
> 65	1.59	1.53–1.65	< 0.0001	1.69	1.62–1.75	< 0.0001
*Chemotherapy*						
No	Reference					
Yes	1.49	1.44–1.55	< 0.0001	0.78	0.74–0.82	< 0.0001
*Grade*						
I	Reference					
II	1.64	1.55–1.73	< 0.0001	1.43	1.35–1.52	< 0.0001
III	2.22	2.1–2.36	< 0.0001	1.66	1.57–1.77	< 0.0001
IV	1.95	1.56–2.43	< 0.0001	1.43	1.15–1.79	0.0016
Unknown	1.44	1.31–1.59	< 0.0001	1.23	1.11–1.35	< 0.0001
*IMA*						
No	Reference					
Yes	0.78	0.66–0.91	0.001	1.03	0.87–1.2	0.7596
*Laterality*						
Left	Reference					
Right	0.97	0.94–1.01	0.113			
*Location*						
Upper	Reference					
Lower	1.1	1.06–1.14	< 0.0001	1.12	1.07–1.16	< 0.0001
Middle	1.08	0.99–1.17	0.08	1.14	1.05–1.24	0.0019
Other	1.55	1.39–1.73	< 0.0001	1.23	1.1–1.37	0.0003
*Marital status*						
Single	Reference					
Divorced	0.93	0.88–0.99	0.016	0.99	0.94–1.05	0.8243
Married	0.85	0.81–0.88	< 0.0001	0.83	0.79–0.86	< 0.0001
*N*						
N0	Reference					
N1	2.11	2.01–2.21	< 0.0001	1.33	1.13–1.57	0.0007
N2	2.44	2.33–2.56	< 0.0001	1.29	0.92–1.81	0.143
N3	3.72	2.87–4.82	< 0.0001	1.88	1–3.55	0.05
*Operation*						
Lobectomy	Reference					
Lobectomy extended	1.46	1.34–1.58	< 0.0001	1.24	1.14–1.35	< 0.0001
Other sublobar	1.29	1.02–1.62	0.031	1.22	0.97–1.54	0.0873
Pneumonectomy	2	1.83–2.18	< 0.0001	1.25	1.14–1.37	< 0.0001
Segmentectomy	1.03	0.92–1.15	0.575	1.2	1.07–1.34	0.0017
Wedge	1.21	1.13–1.29	< 0.0001	1.39	1.3–1.49	< 0.0001
*Race*						
Black	Reference					
Other	0.77	0.7–0.84	< 0.0001	0.76	0.69–0.83	< 0.0001
White	1.02	0.95–1.08	0.636	1.03	0.96–1.1	0.4243
*Radiation*						
No	Reference					
Yes	1.96	1.87–2.06	< 0.0001	1.3	1.22–1.38	< 0.0001
*Sex*						
Male	Reference					
Female	0.69	0.67–0.72	< 0.0001	0.7	0.68–0.73	< 0.0001
Size	1.01	1.01–1.01	< 0.0001	1	1–1	0.0076
*Stage*						
IA1	Reference					
IA2	1.37	1. 22–1.54	< 0.0001	1.28	1.13–1.44	0.0001
IA3	1.87	1.67–2.11	< 0.0001	1.64	1.46–1.86	< 0.0001
IB	2.2	1.94–2.49	< 0.0001	1.71	1.48–1.97	< 0.0001
IIA	2.53	2.21–2.9	< 0.0001	1.91	1.64–2.24	< 0.0001
IIB	3.39	3.01–3.81	< 0.0001	2.43	1.97–3	< 0.0001
IIIA	4.18	3.71–4.7	< 0.0001	2.86	2–4.09	< 0.0001
IIIB	5.71	4.95–6.58	< 0.0001	3.49	1.97–6.18	< 0.0001
IIIC	5.18	2.98–9.03	< 0.0001	2.23	0.81–6.1	0.1186
*T*						
T1	Reference					
TT2	1.54	1.47–1.6	< 0.0001	1.11	1.03–1.19	0.0053
TT3	1.92	1.8–2.05	< 0.0001	0.96	0.79–1.17	0.7066
TT4	2.46	2.27–2.66	< 0.0001	1.08	0.8–1.45	0.6232

**Table 4:** Univariate and multivariate Cox proportional hazards analyses after PSM

Variables	Univariate analysis			Multivariable analysis		
	HR	95% CI	*p*	HR	95% CI	*p*
*Age (years)*						
≤ 65	Reference					
> 65	1.98	1.7–2.31	< 0.0001	1.85	1.57–2.18	< 0.0001
*Chemotherapy*						
No	Reference					
Yes	1.51	1.26–1.8	< 0.0001	0.91	0.73–1.13	0.3975
*Grade*						
I	Reference					
II	1.35	1.12–1.63	0.002	1.17	0.96–1.42	0.1219
III	2.09	1.45–3.01	< 0.0001	1.67	1.12–2.48	0.0116
Unknown	1.1	0.91–1.33	0.329	1.02	0.84–1.24	0.8572
*IMA*						
No	Reference					
Yes	1.04	0.87–1.24	0.652			
*Laterality*						
Left	Reference					
Right	1	0.86–1.15	0.971			
*Location*						
Upper	Reference					
Lower	0.97	0.83–1.13	0.686	1.04	0.89–1.21	0.6488
Middle	0.88	0.62–1.25	0.475	1.06	0.74–1.52	0.7464
Other	1.9	1.21–3	0.006	1.49	0.92–2.41	0.1021
*Marital status*						
Single	Reference					
Divorced	0.77	0.58–1.04	0.087			
Married	0.87	0.73–1.03	0.096			
*N*						
N0	Reference					
N1	1.92	1.38–2.68	< 0.0001	1.04	0.63–1.73	0.8749
N2	2.51	1.82–3.47	< 0.0001	1.06	0.29–3.84	0.9257
*Operation*						
Lobectomy	Reference					
Lobectomy extended	1.67	1.15–2.43	0.007	1.26	0.86–1.86	0.2348
Other sublobar	1.73	0.9–3.34	0.102	1.15	0.57–2.3	0.6961
Pneumonectomy	1.51	0.92–2.49	0.101	0.88	0.52–1.48	0.6188
Segmentectomy	1	0.58–1.74	0.988	1.28	0.74–2.24	0.3779
Wedge	0.95	0.69–1.31	0.752	1.15	0.82–1.6	0.4167
*Race*						
Black	Reference					
Other	0.65	0.44–0.94	0.024	0.54	0.36–0.79	0.0014
White	1.09	0.85–1.39	0.488	0.95	0.74–1.23	0.7218
*Radiation*						
No	Reference					
Yes	2.49	1.84–3.37	< 0.0001	1.54	1.09–2.18	0.0155
*Sex*						
Male	Reference					
Female	0.55	0.48–0.64	< 0.0001	0.64	0.55–0.75	< 0.0001
Size	1.01	1.01–1.01	< 0.0001	1	1–1	0.5011
*Stage*						
IA1	Reference					
IA2	1.59	0.95–2.66	0.08	1.54	0.92–2.59	0.1016
IA3	2.29	1.39–3.76	0.001	2.19	1.32–3.62	0.0024
IB	3.36	2.05–5.51	< 0.0001	2.63	1.13–6.13	0.0245
IIA	3.01	1.79–5.05	< 0.0001	2.32	0.98–5.5	0.0562
IIB	5.43	3.33–8.85	< 0.0001	5.05	2.03–12.59	0.0005
IIIA	7.61	4.68–12.38	< 0.0001	4.94	1.29–18.99	0.0199
IIIB	12.07	6.15–23.7	< 0.0001	10.37	0.97–110.58	0.0527
*T*						
T1	Reference					
T2	1.7	1.41–2.05	< 0.0001	1.17	0.59–2.3	0.658
T3	2.81	2.28–3.46	< 0.0001	0.9	0.41–2.01	0.8048
T4	4.21	3.43–5.18	< 0.0001	1.51	0.44–5.17	0.5135

## Discussion

IMA has unique clinical and pathological features [17–21]. The main difference between IMA, which account for only 5% of all adenocarcinomas, and other lung adenocarcinomas is that the tumor cells consist of goblet cells and columnar cells [4]. IMA was known as mucinous bronchioloalveolar adenocarcinoma until 2011. Although there have been many studies on IMA due to its clinical rarity, the survival of patients with IMA remains controversial, which makes the clinical treatment of IMA difficult. This calls for data analysis from cohorts containing large number of samples, which in turn will further clarify the survival and prognosis of patients with IMA.

Although IMA gene mutations in patients with information from the SEER database is not known, but according to the previously reported [22, 23], KRAS is the most common mutation type, and KRAS mutation may be a poor predictor of survival [24]. This may be because tumors with KRAS mutations may grow faster than tumors without KRAS mutations [25].In addition to KRAS mutation, NRG1 fusion is another common mutation in IMA. It has been reported that 7–27% of IMA have detected NRG1 fusion [23, 26]. EGFR mutations were also found in a small number of IMA patients. Research suggests that EGFR mutation in IMA is an indicator of poor prognosis [27]. Studies indicate abnormal expression of MUC6 in IMA which is associated with survival differences that may reflect MUC6 induced changes in cell adhesion properties [28].

By comparing patients' baseline data, we found that IMA was more likely to occur in the lower lobe, with approximately 59% of patients having tumors in the lower lobe at diagnosis. At the same time, NMA was more likely to occur in the upper lobe, which is in line with the finding of previous studies [11]. The histological grading of IMA was better, in line with the finding of previous studies [13]. Approximately 59% of IMA cases were grade 1 at diagnosis. In contrast, approximately 45% of NMAs were grade II, and 30% were grade III at diagnosis. Contrary to the data of previous studies [29], in our data, there was no difference between the sexes of patients with IMA and NMA. This may be due to the differences between the Eastern and Western populations. In contrast, the T stage, N stage, and pathological stage of the tumor and treatment with radiotherapy showed differences between the IMA group and the NMA group, which is consistent with the results of previous studies [13].

Previous studies have included small sample sizes due to the rarity of IMA. Many studies have reached contradictory conclusions. Some studies found that the OS of patients with IMA is worse than that of patients with NMA [30]. Some studies suggest that the OS of patients with IMA is comparable to that of patients with NMA [31]. Some studies found that the OS of patients with IMA is better than that of patients with NMA [5, 29]. Our data were obtained from the SEER database. A total of 20,914 patients with IMA and NMA were enrolled. PSM was used to minimize the impact of confounding factors on survival outcomes. Survival analysis of patients after PSM showed that the mean survival time of patients with IMA was 124 ± 34 months, with a 3 year survival rate of 73.8% (95% Cl 0.69–0.79). The 5 year survival rate was 66.8% (95% Cl 0.62–0.72). The mean survival in months for patients with NMA was 119 ± 44 months. The 3 year survival rate was 78% (95% CL 0.76–0.80), and the 5 year survival rate was 67.2% (95% CL 0.65–0.70). OS was the same for patients with IMA and NMA (*p* = 0.7). This is consistent with the findings of several previous studies.

After PSM, Cox multivariable analysis of the data showed that patients' OS was not affected by the histological type of the tumor. These results are consistent with those of previous studies [32].Some studies showed that [13] pneumonectomy and sublobar resection can predict OS in patients, which contradicts our findings. This may be due to data discrepancies, and a larger sample size is needed to determine this further. Some studies found that lesion location is also an independent risk predictor of patient OS [29], which is different from our results. This may be due to differences in the data between Eastern and Western populations. Further subgroup analysis of the Eastern and Western populations is needed.

Regarding lymph nodes, patients with IMA and those with NMA showed significant differences, with approximately 94% of patients in the IMA group and approximately 72% of patients in the NMA group being at stage N0. Some previous studies have also shown that patients with IMA have a lower rate of lymph node metastasis [31, 33]. This may show that IMA and NMA are two different types of lung cancer. The results of studies on mutations and immune profiles of both could further confirm this hypothesis [10, 14, 34].

There are limitations to this study. First, due to the small amount of data on stage IV patients, it was not possible to conduct a precise analysis when performing statistical analysis. Therefore, when screening the data, the stage IV data were removed. Only patients with M0 were retained. This made it impossible to compare the survival of stage IV patients. Second, the SEER database only has the OS data of patients and no data on disease recurrence. Therefore, only the OS of patients could be analyzed, not the disease-free survival of patients. A significant indicator for the assessment of patients' condition is missing. Third, the inclusion criteria were based on the patients’ ICD-0-3 code. Although the diagnosis was confirmed by pathology, a small proportion of the pathology was NOS, and this part of the pathology may affect the outcome.

Our article also has some shortcomings, minimally invasive adenocarcinoma (MIA) was diagnosed as invasive adenocarcinoma in version sixth edition TNM stage and not listed separately in the sixth edition TNM stage, but MIA is listed separately in the eighth edition TNM stage, MIA cancer cells also infiltrate, but are listed separately in the eighth edition TNM stage. We examined other SEER database articles [12, 13, 32] which did not specify how to exclude MIA and assume that MIA is also invasive adenocarcinoma.

We matched patients with IMA to patients with NMA by PSM. The impact of confounding factors on the survival of patients in both groups was eliminated as much as possible. However, due to the rarity of IMA, although patients diagnosed from 2000 to 2014 were selected, only 303 patients with IMA met the criteria. We also attempted to screen the data for patients diagnosed in 2015–2019, but unfortunately, due to the revision of the SEER database and the lack of various patient information, it was impossible to filter the required patient information from 2015 to 2019. This also greatly limited the number of cases enrolled. Finally, as this study was retrospective, and compared to prospective studies with the highest level of evidence, there were inevitable confounding factors despite PSM. Despite these limitations, the present study has merit. We selected data from 17 centers in the SEER database to avoid bias due to single-center data. Second, the data collected had information on patients' radiotherapy and chemotherapy, allowing us to assess the sensitivity of patients with IMA and NMA to radiotherapy and chemotherapy.

## Conclusion

In conclusion, there was no significant difference in OS between the pathological subtypes of IMA and NMA in patients with a pathological staging of M0. Age, gender, and radiation therapy receipt independently predicted patient OS. Our findings can be combined with those of previous studies to jointly analyze survival in patients with IMA.

## Data Availability

The datasets generated and/or analyzed during the current study are available in the SEER database. The address of the SEER database is: https://seer.cancer.gov/. The data that support the findings of this study are openly available in [SEER database].
